# Feasibility of the Manchester Acute Coronary Syndromes (MACS) decision rule to safely reduce unnecessary hospital admissions: a pilot randomised controlled trial

**DOI:** 10.1136/emermed-2016-206148

**Published:** 2017-05-12

**Authors:** Richard Body, Charles Boachie, Alex McConnachie, Simon Carley, Patricia Van Den Berg, Fiona E Lecky

**Affiliations:** 1 Cardiovascular Sciences, The University of Manchester, Manchester, UK; 2 Emergency Department, Central Manchester University Hospitals Foundation NHS Trust, Manchester, UK; 3 Robertson Centre for Biostatistics, Institute of Health and Wellbeing, University of Glasgow, Glasgow, UK; 4 Healthcare Sciences, Manchester Metropolitan University, Manchester, UK; 5 School of Medicine, Maastricht University, Maastricht, The Netherlands; 6 Emergency Department, Salford Royal Infirmary, Manchester, UK

**Keywords:** Acute myocardial infarction, Acute Coronary Syndromes, Diagnosis, Sensitivity and Specificity, Troponins, Troponins, high sensitivity, Emergency Medicine, Clinical Decision Rules

## Abstract

**Background:**

Observational studies suggest that the Manchester Acute Coronary Syndromes (MACS) decision rule can effectively ‘rule out’ and ‘rule in’ acute coronary syndromes (ACS) following a single blood test. In a pilot randomised controlled trial, we aimed to determine whether a large trial is feasible.

**Methods:**

Patients presenting to two EDs with suspected cardiac chest pain were randomised to receive care guided by the MACS decision rule (intervention group) or standard care (controls). The primary efficacy outcome was a successful discharge from the ED, defined as a decision to discharge within 4 hours of arrival providing that the patient did not have a missed acute myocardial infarction (AMI) or develop a major adverse cardiac event (MACE: death, AMI or coronary revascularisation) within 30 days. Feasibility outcomes included recruitment and attrition rates.

**Results:**

In total, 138 patients were included between October 2013 and October 2014, of whom 131 (95%) were randomised (66 to intervention and 65 controls). Nine (7%) patients had prevalent AMI and six (5%) had incident MACE within 30 days. All 131 patients completed 30-day follow-up and were included in the final analysis with no missing data for the primary analyses. Compared with standard care, a significantly greater proportion of patients whose care was guided by the MACS rule were successfully discharged within 4 hours (26% vs 8%, adjusted OR 5.45, 95% CI 1.73 to 17.11, p=0.004). No patients in either group who were discharged within 4 hours had a diagnosis of AMI or incident MACE within 30 days (0.0%, 95% CI 0% to 20.0% in the intervention group).

**Conclusions:**

In this pilot trial, use of the MACS rule led to a significant increase in safe discharges from the ED but a larger, fully powered trial remains necessary. Our findings seem to support the feasibility of that trial.

**Trial registration number:**

ISRCTN 86818215.

**Research Ethics Committee reference:**

13/NW/0081.

**UKCRN registration ID:**

14334.

**Key messages**

**What is already known on this subject?**

- Observational research has shown that the Manchester Acute Coronary Syndromes (MACS) decision rule could be used to safely reduce hospital admissions and make judicious use of inpatient resources by ‘ruling out’ and ‘ruling in’ acute coronary syndromes following a single blood test in the ED. We do not yet know how the MACS rule will perform when used to guide the management of patients in real-life practice.

**What this study adds?**

- Our findings seem to suggest that a larger trial will be feasible to conduct, although we will need to ensure adequate support to achieve a sufficient recruitment rate at each trial centre. In this small pilot randomised controlled trial, patients whose care was guided by the MACS rule were more likely to be successfully discharged within 4 hours of arrival than patients receiving standard care. None of those patients had a major adverse cardiac event within 30 days (part of the primary efficacy outcome) or within 6 months (secondary outcome), although a larger trial is required to provide greater statistical power for that analysis.

## Background

Current approaches to ‘ruling out’ acute coronary syndromes (ACS) rely on serial biomarker testing over a number of hours. The Manchester Acute Coronary Syndromes (MACS) decision rule is designed to enable clinicians in the ED to ‘rule in’ and ‘rule out’ ACS following a single blood test.^1 2^ The MACS rule, which was derived by logistic regression, incorporates eight variables (table 1). The rule estimates the probability that a patient either has AMI or will develop a major adverse cardiac event (MACE) within 30 days. Based on that probability, patients are assigned to one of four risk groups, each of which has a recommended destination for the patient: ‘very low risk’ (home); ‘low risk’ (low dependency observation ward); ‘moderate risk’ (acute inpatient area; intermediate dependency); and ‘high risk’ (specialist cardiology ward or high-dependency environment). In two external validation studies including a total of 1245 patients, the MACS rule successfully risk stratified patients and identified a group potentially suitable for immediate discharge.^1 3^ While there are also other promising early ‘rule out’ strategies, we chose to evaluate MACS as it can both ‘rule in’ and ‘rule out’ ACS with a single blood test at the time of arrival in the ED.

**Table 1:** Components of the MACS decision rule

**Variable**	**Format**
a. High-sensitivity cardiac troponin T (ng/L)	Continuous variable
b. Heart type fatty acid binding protein (ng/mL)	Continuous variable
c. Acute ECG ischaemia (treating clinician’s interpretation)	Dichotomous
d. Sweating observed by the treating clinician	Dichotomous
e. Vomiting in association with the presenting symptoms	Dichotomous
f. Systolic BP <100 mm Hg on arrival	Dichotomous
g. Worsening (or crescendo) angina	Dichotomous
h. Pain radiating to the right arm or shoulder	Dichotomous

The MACS rule estimates the probability (p) of acute coronary syndromes as follows (rounded values are presented): p=1/(1+e-^(0.068a + (0.17(b -0.28)/1.35) + 1.75c + 1.85d + 1.72e + 1.46f + 0.92g + 0.87h -4.83)^). For dichotomous variables, a value of ‘1’ is entered for ‘yes’ and ‘0’ for ‘no’. The constants presented here assume use of the Roche Elecsys hs-cTnT assay and the Randox Laboratories immunoturbidimetric H-FABP assay.

H-FABP, heart type fatty acid binding protein; hs-cTnT, high sensitivity cardiac troponin T; MACS, Manchester Acute Coronary Syndrome.

Evaluating new diagnostic technology in observational research alone has important limitations. It is possible that any beneficial effects will be diluted when rules are implemented in practice because clinicians do not abide by the recommendations. Unanticipated effects, such as rebound overuse of resources in patients who cannot have ACS ‘ruled out’, have previously been reported and have meant that apparently safe pathways are not cost-effective.^4^ The next phase in the evaluation of the MACS rule is therefore to evaluate its impact in clinical practice. This can be robustly accomplished in a randomised controlled trial (RCT). The value of a rigorous feasibility trial, in which trial design and procedures are evaluated before embarking on a full-scale trial, is increasingly recognised.

We therefore aimed to evaluate the feasibility of running a multicentre RCT to compare the use of the MACS rule to standard practice. While recognising that the analysis was likely to be underpowered in this pilot trial, we also sought to evaluate the efficacy of the MACS rule as a tool to increase the proportion of patients successfully discharged within 4 hours of arrival in the ED.

## Methods

### Design and setting

We conducted a prospective, pragmatic, pilot RCT at two centres in Greater Manchester, UK (Manchester Royal Infirmary and Salford Royal Infirmary). Written informed consent was obtained from all participants and the study was approved by the research ethics committee (reference 13/NW/0081).

### Participants

We included adults (>18 years) presenting to the ED with pain, discomfort or pressure in the chest, epigastrium, neck, jaw or upper limb without an apparent non-cardiac source (compatible with the American Heart Association case definitions,^5^ which the treating physician believed warranted investigation for possible ACS. We excluded patients with peak symptoms occurring >24 hours prior to presentation; those who could not have been discharged if ACS had been ruled out; patients with definite ST elevation myocardial infarction; those with no capacity to provide written informed consent; those unable to communicate in English language unless translation services were available; and prisoners.

### Intervention and randomisation

Consenting participants were randomly allocated to the intervention or control group in a 1:1 ratio using a central, Internet-based randomisation service managed by Glasgow Clinical Trials Unit. Randomisation was stratified by trial centre and MACS rule risk group. Admission blood samples were tested for high sensitivity cardiac troponin T (hs-cTnT; Roche Diagnostics Elecsys, 99th percentile 14 ng/L, coefficient of variation <10% at 13 ng/L) and heart type fatty acid binding protein (H-FABP; Randox Laboratories immunoturbidimetric assay). Clinical data were recorded contemporaneously by the treating clinician using a bespoke case report form, and later transferred to an electronic case report form by research nurses or investigators.

In both groups, the required data for calculation of the MACS rule outcome were entered prior to randomisation using an online tool created by Glasgow Clinical Trials Unit. To avoid contamination between trial groups, the outcome of the MACS rule calculation was only revealed for patients randomised to the intervention group. Patients randomised to this group had their care guided by the MACS decision rule. The website calculated the estimated probability of MACE, stratified patients into four risk groups and recommended the following disposition for patients: ‘very low risk’ patients could be discharged; ‘low risk’ patients should undergo serial troponin testing in either an ED observation ward or (if that is not available) an acute medical unit; ‘moderate risk’ patients should undergo serial troponin testing in an acute medical unit; and ‘high-risk’ patients should be referred to a cardiologist and managed in a specialist or high-dependency environment. As part of the follow-up for this trial, all participants who were discharged from the ED without hs-cTnT sampling at least 12 hours after peak symptoms were given a follow-up appointment within 72 hours, at which point hs-cTnT concentration was again measured. This ensured that we were able to detect any missed prevalent AMIs in this group.

Care of the control group was guided by local ED guidelines for the management of suspected acute coronary syndromes, which were consistent with contemporaneous national guidance.^6^ At both trial centres, this required measuring hs-cTnT on arrival and 12 hours after peak symptoms.

In this pragmatic trial, patients in both groups who presented >12 hours after peak symptoms could have AMI ‘ruled out’ using a single hs-cTnT concentration, in accordance with contemporary national guidance at the time of the trial.^6^ This meant that, in both groups, clinicians could discharge late presenting patients following a single troponin test if deemed clinically appropriate. As an example, a patient in the intervention group could have been identified as being at ‘moderate risk’ with a suggestion for serial troponin testing in an acute medical ward. If, however, the admission troponin sample was drawn >12 hours after peak symptoms, the need for serial troponin sampling was obviated, and the patient could still be discharged if the treating clinician deemed this to be clinically appropriate.

### Follow-up

Participants were followed up after 30 days, 3 months and 6 months by electronic chart review and either telephone, email or letter. At each visit, participants completed a structured questionnaire to determine clinical events, healthcare resource use and EQ-5D. Each participant was also asked to complete a modified Group Health Association of America (GHAA) questionnaire to assess patient satisfaction after 30 days and 6 months.

### Outcomes

The primary efficacy outcome was successful early discharge, defined as a decision to discharge the patient from hospital within 4 hours of arrival (specified a priori and based on the process target within the UK), excluding those patients who either (1) had a missed diagnosis of AMI or (2) experienced MACE within 30 days. MACE included death (all cause), incident AMI or coronary revascularisation procedures. Secondary outcomes included^1^: the incidence of MACE after 30 days, 3 months and 6 months and^2^ length of initial hospital stay.

Feasibility outcomes included: (1) the number of eligible patients approached; (2) the proportion of approached patients randomised; (3) attrition (including both failure to complete the trial protocol and loss to follow-up); (4) completeness of data collection; and (5) patient satisfaction. We also further explored the feasibility of the trial processes to clinicians and participants using qualitative methods, which we plan to report separately.

### Measuring outcomes

The dates and times of admission, decision to discharge and actual discharge were determined from electronic hospital records. The time to decision to discharge was calculated as the time between arrival in the ED and the time of actual discharge unless it had been documented that the patient was medically fit for discharge at an earlier stage but discharge was delayed pending other factors (for example, waiting for transport home). The case report form, which was completed by the treating clinician, asked clinicians to record the time of this decision.

Death and coronary revascularisation were determined at follow-up from review of a national mortality database (NHS Spine), hospital records (including any relevant investigation or procedure reports) and patient self-report. The diagnosis of AMI was adjudicated by two independent investigators in accordance with the third universal definition of myocardial infarction^7^ and with reference to all relevant investigations (including the ECG, cardiac troponin concentrations and follow-up data including the findings of any relevant imaging investigations) but blinded to the MACS rule. In order to fulfil the diagnosis of AMI, patients were required to have a rise and/or fall of hs-cTnT with at least one level above the 99^th^ percentile (14 ng/L). An absolute change of 9.2 ng/L on serial hs-cTnT sampling was deemed to demonstrate a rise and/or fall.^8^ Of note, to avoid verification bias, reference standard late troponin testing was undertaken in all patients. Patients who were discharged early as part of this trial still underwent late troponin sampling as detailed above, on an outpatient basis. This enabled us to be conf ident about the prevalence of missed AMI in the trial. The patient group we consulted when designing this trial fed back that this element of the design was important and desirable.￼

Patient satisfaction was measured at follow-up after 30 days using a modified GHAA consumer satisfaction survey.^9^ The number of eligible patients approached was determined from contemporaneous screening records maintained by research staff.

### Statistical analysis

All statistical analyses were specified in the protocol and described in a detailed statistical analysis plan prior to the final unblinded analysis, which was undertaken by the trial statisticians (AM and CB). We calculated the OR for achie ving the primary efficacy outcome by logistic regression with the primary outcome as the dependent variable. An initial binary logistic regression analysis was undertaken to yield a simple unadjusted OR with a 95% CI. For the primary analysis (from which conclusions are drawn), important covariates were also entered into the model (age, gender, trial centre, MACS risk group, cardiovascular risk factors and a prior history of coronary artery disease). Similarly, the absolute rate of MACE (secondary outcome) at 30 days, 3 months and 6 months was compared between groups using logistic regression. Length of stay and patient satisfaction data were compared between randomised groups using the Mann-Whitney U test. Finally, we calculated the incidence of MACE at 30 days, 90 days and 180 days stratified by MACS rule risk group in all participants. Statistical analyses were undertaken using SAS for Windows V.9.3. As this is a feasibility study, no formal sample size calculation was undertaken but we aimed to evaluate recruitment rate over a fixed period.

## Results

**Figure 1:**
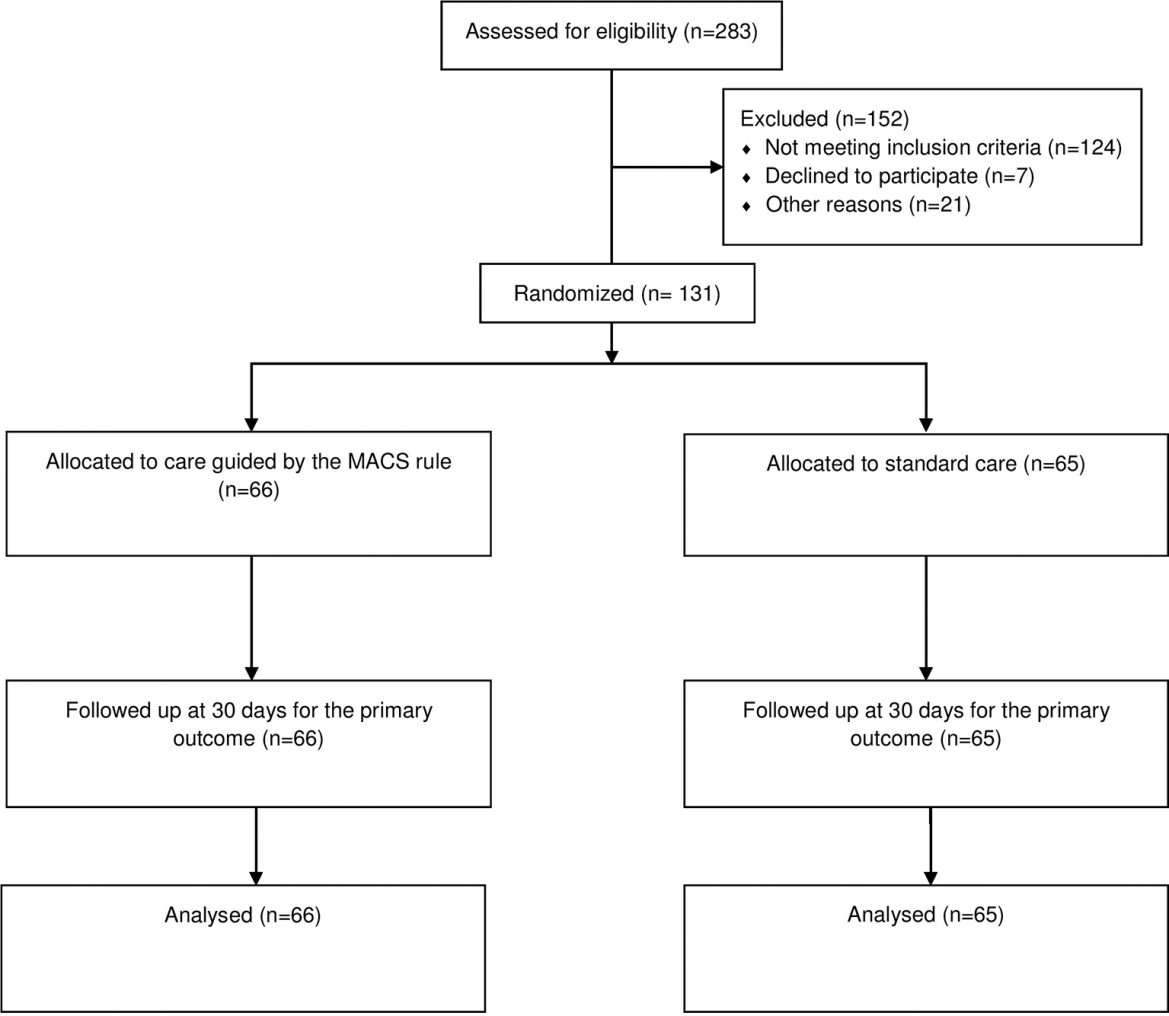
Participant flow diagram.

Of 283 patients screened, 138 were eligible and included between 29 October 2013 and 24 January 2014 (Manchester) and between 24 July 2014 and 10 October 2014 (Salford). Figure 1 shows a participant flow diagram detailing the patients screened, eligible, included, randomised and followed up. Baseline characteristics of participants are shown in table 2.

**Table 2:** Baseline characteristics of participants

**Characteristics**		**All, n=131**	**Intervention group, n=66**	**Control group, n=65**
Centre, n (%)	Central Manchester￼ Salford	113 (86) 18 (14)	57 (86) 9 (14)	56 (86) 9 (14)
Age, mean (SD)		58.9 (16.3)	60.8 (14.8)	56.9 (17.5)
Female sex, n (%)		52 (40)	28 (42)	24 (37)
Ethnic origin, n (%)	British White	87 (66)	43 (65)	44 (68)
	Asian Pakistani	12 (9)	5 (8)	7 (11)
	Asian Other	7 (5)	5 (8)	7 (11)
	Irish White	5 (4)	3 (5)	2 (3)
	Black Caribbean	4 (3)	2 (3)	2 (3)
	Other	12 (9)	7 (11)	5 (8)
	Does not wish to answer	4 (3)	1 (2)	3 (5)
Previous myocardial infarction, n (%)		44 (34)	20 (30)	24 (37)
Previous history of angina, n (%)		47 (36)	25 (38)	22 (34)
Previous percutaneous coronary intervention, n (%)		31 (24)	18 (27)	13 (20)
Previous coronary artery bypass graft, n (%)		7 (5)	2 (3)	5 (8)
Previous history of hypertension, n (%)		56 (43)	29 (44)	27 (42)
Previous history of hyperlipidaemia, n (%)		44 (34)	23 (35)	21 (32)
Type 1 diabetes, n (%)		2 (2)	1 (2)	1 (2)
Type 2 diabetes, n (%)		24 (18)	14 (21)	10 (15)
Median time from symptom onset (IQR)		45.00 (15.00 to 120.00)	35.00 (10.00 to 150.00)	60.00 (20.00 to 120.00)
MACS rule risk group, n (%)	Very low risk	33 (25)	16 (24)	17 (26)
	Low risk	36 (27)	18 (27)	18 (28)
	Moderate risk	57 (44)	29 (44)	28 (43)
	High risk	5 (4)	3 (5)	2 (3)

### Feasibility outcomes

Of the 138 included patients, 131 (95%) were randomised and included in the analysis. The data were 100.0% complete for all randomised patients with regard to the variables incorporated in the MACS rule, patient symptoms, history, physical examination, clinician gestalt, disposition from the ED and follow-up for clinical events after 30 days. All patients had sufficient follow-up data for evaluation of the primary outcome at 30 days. A total of 119 (91%) patients completed the patient satisfaction questionnaire after 30 days and there were no significant differences in patient satisfaction between the groups (table 3 and online supplementary table 1). At 6 months, 107 (82%) patients had completed follow-up.

10.1136/emermed-2016-206148.supp1 Supplementary appendix

**Table 3:** Incidence of the primary and secondary efficacy outcomes stratified by trial group

Outcome	Total n=131	Intervention n=66	Control n=65		OR (95% CI),p value
Early discharge within 4 hour without prevalent AMI or incident MACE at 30 days, n (% (95% CI))	22 (17%)	17 (26%)	5 (8%)	Unadjusted Adjusted*	4.16 (1.43 to 12.09), p0.009 5.45 (1.73 to 17.11), p=0.004
Prevalent AMI, n (%)	9 (7)	3 (5)	6 (9)		0.47 (0.11 to 1.96), p=0.30
Incident MACE at 30 days†, n (%)	6 (5)	3 (5)	3 (5)		0.98 (0.19 to 5.06), p=0.985
Incident MACE at 90 days†, n (%)	9 (7)	5 (8)	4 (6)		1.36 (0.35 to 5.34), p=0.656
Incident MACE at 180 days†, n (%)	11 (8)	6 (9)	5 (8)		1.30 (0.37 to4.56), p=0.678
Hospital length of stay (median, IQR)	1 (0–1)	1 (0–1)	1 (0–1)		p=0.54‡
Patient satisfaction (mean score, SD)	3.8 (1.0)	3.8 (1.0)	3.8 (1.1)		p=0.80‡
Re-attendance at an ED, n (%)	18 (23)	10 (23)	8 (23)		p=0.97
Any further investigation for heart disease within 30 days, n (%)	27 (21)	15 (23)	12 (18)		p=0.55
Myocardial perfusion imaging (thallium scan) within 30 days, n (%)	8 (28)	3 (19)	5 (38)		p=0.24
Stress ECG within 30 days, n (%)	1 (4)	1 (7)	0 (0)		p=0.34
Coronary angiography within 30 days, n (%)	9 (32)	4 (27)	5 (38)		p=0.51

*Adjusted for trial centre, age, gender, cardiovascular risk factors and history of prior coronary artery disease. This is the prespecified primary analysis.

†Incident death, AMI or coronary revascularisation (not including prevalent AMI). NB, no patients who were discharged early developed a MACE.

**‡**Mann-Whitney U test.

MACE, major adverse cardiac event.

### Primary efficacy outcome

In total, 17 (26%) patients in the intervention group met the primary outcome of successful discharge from the ED within 4 hours without MACE after 30 days compared with 5 (8%) patients in the control group. This yielded an unadjusted OR of 4.16 (95% CI 4.16 to 12.09, p=0.009) and an adjusted OR of 5.45 (95% CI 1.73 to 17.11, p=0.004), indicating that a significantly greater proportion of patients in the intervention group achieved the primary outcome. None of the patients in either group who were discharged within 4 hours had a diagnosis of AMI or incident MACE within 30 days. Thus, in the intervention group, 0 (0.0%, 95% CI 0% to 20.0%) patients who were discharged from the ED developed MACE within 30 days.

In the intervention group, 16 (24.2%) patients had been identified as being at ‘very low risk’ by the MACS rule, of whom 12 (75.0%) were actually discharged within 4 hours of arrival. One patient in the ‘very low risk’ group was discharged directly from the ED but did not meet the primary outcome as the time of discharge was recorded as being 4.4 hours after arrival. Of the three remaining ‘very low risk’ patients in the intervention group who were not discharged within 4 hours, in two cases this was caused by a delay to obtaining H-FABP results from the laboratory (both patients were sent to in patient areas to await results). In the remaining case, the patient had been discharged home within 4 hours of arrival but an electronic hospital system had recorded the disposition as the ED observation ward rather than ‘home’. While this appears to have been an administrative error, we made a conservative assumption that the patient had not been discharged directly from the ED and therefore did not meet the primary outcome.

Five (7.6%) patients in the intervention group were discharged from the ED despite not being in the ‘very low risk’ group, three of whom were ‘low risk’ and two of whom were ‘moderate risk’ according to the MACS rule. All of these patients had presented late (>12 hour) after symptom onset. While this remains entirely in accordance with acceptable practice during the study period, we ran a conservative sensitivity analysis considering that patients in the intervention group who were discharged early despite not being in the ‘very low’ risk group did not meet the primary outcome. With this assumption, the proportion of patients meeting the primary outcome of successful discharge remained significantly higher in the intervention group (12 (18%) vs 2 (3%), OR 7.0, 95% CI 1.5 to 32.7, p=0.013).

### Secondary efficacy outcomes

Noting the important fact that no formal power calculation had been conducted and that this pilot trial included a relatively small number of patients, we did not detect any significant differences in the incidence of MACE at 30 days (5% vs 5%, p=0.985), 3 months (8% vs 6%, p=0.656) or 6 months (9% vs 8%, p=0.678) between groups (table 3). Similarly, we did not detect any apparent differences in length of hospital stay, ED re-attendance or further investigation for coronary artery disease (summarised in table 3). The incidence of MACE stratified by MACS rule risk group among all patients is shown in Table 4. Among patients in the MACS rule ‘very low risk group’, the mean length of stay was 2.0 hours (SD 6.9 hours) in the intervention group versus 10.5 hours (SD 12.3 hours) in the control group (see online supplementary table 2).

**Table 4:** Incidence of MACE stratified by MACS rule risk group in all patients

	Very low risk	Low risk	Moderate risk	High risk
Total number of patients (%) in group	33 (25.2)	36 (27.5)	57 (43.5)	5 (3.8)
MACE at 30 days, n (%)	0 (0)	0 (0)	4 (7)	2 (40)
MACE at 90 days, n (%)	0 (0)	1 (3)	6 (11)	2 (40)
MACE at 180 days, n (%)	0 (0)	1 (4)	8 (18)	2 (40)

MACE, major adverse cardiac event; MACS, Manchester Acute Coronary Syndromes.

## Discussion

A number of our findings tend to suggest that a larger multicentre RCT will be feasible. For example, this study has demonstrated that a satisfactory proportion of patients proceeded to randomisation (95%) and completed 30-day follow-up for evaluation of the primary outcome (100%). We observed no missing data for important variables. The recruitment rate at the Manchester site (120 participants in 12 weeks) met the prespecified expected rate of 10 participants per week. We have, however, detected issues that will need to be addressed to optimise delivery of such a trial. The recruitment rate at the Salford site was much slower than anticipated (18 participants in 11 weeks or 1.6 participants per week). The reasons for this discrepancy will be explored through qualitative analyses that will be presented separately.

In this pilot trial, we also found that the use of the MACS rule in real-life clinical settings led to a significant reduction in unnecessary hospital admissions. Over one quarter (26%) of patients in the intervention group were successfully discharged without a missed AMI or incident MACE within 30 days, compared with 8% in the control group, which was highly statistically significant (p=0.004).

The detection of a significant increase in the proportion of patients successfully discharged within 4 hours of arrival in the ED, however, is unlikely to be sufficient to change practice. Clinicians are likely to require robust evidence that the incidence of MACE is sufficiently low in discharged patients to change their practice. In this pilot trial, the upper bound of the 95% CI for the incidence of MACE among discharged patients in this trial was as high as 20%. A larger trial is therefore still required to obtain a more precise estimate of the incidence of MACE among patients who are discharged early.

### Limitations

Aside from the requirement for a larger study, it is important to note that the MACS rule has to date been validated with one troponin assay (hs-cTnT) and incorporates H-FABP, which is not currently used in routine clinical practice. There remains a need for published validation of MACS with different troponin assays. Evaluation of the recently refined ‘troponin-only MACS rule’, which does not require H-FABP measurement, is also still required.^10^

### Future directions

In this rapidly changing field, it is now prudent to consider the appropriate comparator for a definitive RCT of the MACS rule. In our study, standard care included cardiac troponin testing 12 hours after symptom onset, whereas new national recommendations allow for AMI to be ‘ruled out’ with two blood tests taken 3 hours apart, regardless of the time from symptom onset.^11^ As there is a 4-hour process target in the UK, it is unlikely that use of this comparator would lead to different results than we observed. However, there are also emerging alternatives to the MACS rule that could ‘rule out’ ACS in some patients with a single blood test. The HEART score (History, ECG, Age, Risk Factors, Troponin), for example, uses elements of the patient’s history, ECG findings, age, risk factors for coronary artery disease and initial coronary artery disease to identify a group of patients that could be immediately discharged.^12^ AMI could potentially also be ‘ruled out’ in patients with hs-cTnT concentrations below the limit of detection of the assay and no ECG ischaemia.^13–16^ Other tools, such as the Emergency Department Assessment of Chest Pain Score (ED-ACS) or bespoke algorithms to rule out AMI with hs-cTnT testing alone, could be used to discharge more patients following serial troponin testing after 1 to 2 hours.^17 18^ Some of these alternatives have already been evaluated in RCTs. Two-hour troponin sampling used alongside the thrombolysis in myocardial infarction risk score has been shown to increase the proportion of patients discharged from the ED within 6 hours compared with standard care.^19^ A subsequent trial showed that use of the ED-ACS score could achieve a similar proportion of early discharges.^17^ Three-hour troponin sampling used alongside the HEART score has also been shown to increase the proportion of early discharges in a trial from the USA.^20^

With several potential comparators, it seems prudent to undertake direct comparisons of the diagnostic accuracy of alternative strategies, which may be most efficiently achieved through observational studies. The findings of such head-to-head comparisons would then inform the design of future large trials.

A RCT remains important as the MACS rule may have important advantages over other strategies. For example, MACS (1) does not rely on serial blood sampling, (2) takes account of clinical information (such as the nature of a patient’s symptoms and ECG findings), which clinicians are unlikely to ignore in practice, (3) risk stratifies undifferentiated patients with possible cardiac chest pain without the need for prior risk stratification (and could thus help to both ‘rule in’ and ‘rule out’ ACS) and (4) calculates the probability of ACS as a percentage. Future work could explore whether this function is informative and constructive for clinicians and whether it could be used as part of a shared decision-making approach, which has previously been shown to be effective in this patient group.^21^

## Conclusions

Based on the observed recruitment rate, randomisation, data completeness and attrition, our findings seem to support the feasibility of a large trial comparing the MACS rule to standard care, although recruitment rate was suboptimal at one of our two trial sites. In this pilot trial, the use of the MACS decision rule also led to a greater proportion of patients being successfully discharged from the ED within 4 hours of arrival. However, a larger trial is still required to provide more precise estimates of safety outcomes.
