# Supplementary material for: Feasibility of the Manchester Acute Coronary Syndromes (MACS) decision rule to safely reduce unnecessary hospital admissions: a pilot randomised controlled trial
Source: Emerg Med J. 2017 May 12;34(9):586–92. doi: 10.1136/emermed-2016-206148 (PMC5574380; doi:10.1136/emermed-2016-206148)
Supplement: Supplementary appendix [file emermed-2016-206148supp001.pdf]

Appendix

Supplementary Table 1: Findings of the patient satisfaction questionnaire administered at 30 days

Characteristics	.	All N = 127	Treatment N = 66	Control N = 61	p-value
**The urgency with which you were assessed**	N (non-missing)	115	57	58	0.80
	Missing (%, [95% CI])	12 (9 %, [5 to 16])	9 (14 %, [6 to 24])	3 (5 %, [1 to 14])	
	Mean (SD)	3.89 (0.96)	3.88 (0.89)	3.90 (1.04)	
	Median (IQR)	4.00 (3.00 to 5.00)	4.00 (3.00 to 5.00)	4.00 (3.00 to 5.00)	
	Min - Max	1.00 - 5.00	2.00 - 5.00	1.00 - 5.00	
**The thoroughness of your assessment**	N (non-missing)	115	57	58	0.41
	Missing (%, [95% CI])	12 (9 %, [5 to 16])	9 (14 %, [6 to 24])	3 (5 %, [1 to 14])	
	Mean (SD)	3.83 (1.01)	3.77 (0.91)	3.88 (1.11)	
	Median (IQR)	4.00 (3.00 to 5.00)	4.00 (3.00 to 4.00)	4.00 (3.00 to 5.00)	
	Min - Max	1.00 - 5.00	2.00 - 5.00	1.00 - 5.00	
**Explanations given to you about medical procedures & tests**	N (non-missing)	115	57	58	0.34
	Missing (%, [95% CI])	12 (9 %, [5 to 16])	9 (14 %, [6 to 24])	3 (5 %, [1 to 14])	
	Mean (SD)	3.58 (1.13)	3.47 (1.15)	3.69 (1.11)	
	Median (IQR)	4.00 (3.00 to 5.00)	3.00 (3.00 to 4.00)	4.00 (3.00 to 5.00)	
	Min - Max	1.00 - 5.00	1.00 - 5.00	1.00 - 5.00	
**Attention given to what you have to say**	N (non-missing)	115	57	58	0.22
	Missing (%, [95% CI])	12 (9 %, [5 to 16])	9 (14 %, [6 to 24])	3 (5 %, [1 to 14])	
	Mean (SD)	3.60 (1.11)	3.47 (1.14)	3.72 (1.09)	
	Median (IQR)	4.00 (3.00 to 5.00)	3.00 (3.00 to 4.00)	4.00 (3.00 to 5.00)	
	Min - Max	1.00 - 5.00	1.00 - 5.00	1.00 - 5.00	
**Advice you got about ways to avoid illness and stay healthy**	N (non-missing)	115	57	58	0.07
	Missing (%, [95% CI])	12 (9 %, [5 to 16])	9 (14 %, [6 to 24])	3 (5 %, [1 to 14])	

Characteristics	.	All N = 127	Treatment N = 66	Control N = 61	p-value
	Mean (SD)	2.78 (1.24)	2.98 (1.14)	2.59 (1.31)	
	Median (IQR)	3.00 (2.00 to 4.00)	3.00 (2.00 to 4.00)	3.00 (1.00 to 3.00)	
	Min - Max	1.00 - 5.00	1.00 - 5.00	1.00 - 5.00	
**Friendliness & courtesy shown to you by hospital staff**	N (non-missing)	115	57	58	0.79
	Missing (%, [95% CI])	12 (9 %, [5 to 16])	9 (14 %, [6 to 24])	3 (5 %, [1 to 14])	
	Mean (SD)	4.01 (0.92)	4.00 (0.87)	4.02 (0.98)	
	Median (IQR)	4.00 (3.00 to 5.00)	4.00 (3.00 to 5.00)	4.00 (3.00 to 5.00)	
	Min - Max	1.00 - 5.00	2.00 - 5.00	1.00 - 5.00	
**Personal interest in you & your medical problems**	N (non-missing)	115	57	58	0.40
	Missing (%, [95% CI])	12 (9 %, [5 to 16])	9 (14 %, [6 to 24])	3 (5 %, [1 to 14])	
	Mean (SD)	3.61 (1.03)	3.54 (0.96)	3.67 (1.10)	
	Median (IQR)	4.00 (3.00 to 4.00)	3.00 (3.00 to 4.00)	4.00 (3.00 to 5.00)	
	Min - Max	1.00 - 5.00	1.00 - 5.00	1.00 - 5.00	
**Respect shown to you, and attention to your privacy**	N (non-missing)	115	57	58	0.41
	Missing (%, [95% CI])	12 (9 %, [5 to 16])	9 (14 %, [6 to 24])	3 (5 %, [1 to 14])	
	Mean (SD)	3.81 (0.93)	3.75 (0.83)	3.86 (1.02)	
	Median (IQR)	4.00 (3.00 to 5.00)	4.00 (3.00 to 4.00)	4.00 (3.00 to 5.00)	
	Min - Max	1.00 - 5.00	2.00 - 5.00	1.00 - 5.00	
**Reassurance & support offered to you by hospital staff**	N (non-missing)	115	57	58	0.88
	Missing (%, [95% CI])	12 (9 %, [5 to 16])	9 (14 %, [6 to 24])	3 (5 %, [1 to 14])	
	Mean (SD)	3.51 (1.10)	3.53 (1.10)	3.50 (1.11)	
	Median (IQR)	3.00 (3.00 to 4.00)	3.00 (3.00 to 4.00)	3.50 (3.00 to 4.00)	
	Min - Max	1.00 - 5.00	1.00 - 5.00	1.00 - 5.00	
**Amount of time the hospital staff gave you**	N (non-missing)	115	57	58	0.77
	Missing (%, [95% CI])	12 (9 %, [5 to 16])	9 (14 %, [6 to 24])	3 (5 %, [1 to 14])	
	Mean (SD)	3.58 (1.12)	3.61 (1.10)	3.55 (1.14)	

Characteristics	.	All<br>N = 127	Treatment<br>N = 66	Control<br>N = 61	p-value
	Median (IQR)	4.00 (3.00 to 5.00)	4.00 (3.00 to 5.00)	3.50 (3.00 to 5.00)	
	Min - Max	1.00 - 5.00	1.00 - 5.00	1.00 - 5.00	
**Overall, how satisfied are you by the service you received**	N (non-missing)	115	57	58	0.80
	Missing (%, [95% CI])	12 (9 %, [5 to 16])	9 (14 %, [6 to 24])	3 (5 %, [1 to 14])	
	Mean (SD)	3.78 (1.03)	3.77 (0.96)	3.79 (1.10)	
	Median (IQR)	4.00 (3.00 to 5.00)	4.00 (3.00 to 5.00)	4.00 (3.00 to 5.00)	
	Min - Max	1.00 - 5.00	2.00 - 5.00	1.00 - 5.00	

Supplementary Table 2: Total length of stay in hospital (hours) by MACS risk group.

MACS rule risk group	Number of patients admitted to hospital from the ED / Total<br>N=123	Mean (SD) length of stay in hours: all patients	Intervention group: Number of patients admitted to hospital / Total<br>N= 60	Intervention group: mean (SD) length of stay in hours	Control group: Number of patients admitted to hospital / Total<br>N=63	Control group: mean (SD) length of stay in hours
Very low risk	8/28 (29%)	6.9 (11.04)	1/12 (8%)	2.0 (6.93)	7/16(44%)	10.5 (12.30)
Low risk	18/35 (51%)	17.8 (26.25)	7/18 (39%)	14.7 (28.68)	11/17 (65%)	21.2 (23.82)
Medium risk	40/56 (71%)	52.3 (73.67)	22/28 (79%)	42.0 (49.58)	18/28 (64%)	62.6 (91.53)
High risk	4/4 (100%)	474.0 (345.79)	2/2 (100%)	276.0 (84.85)	2/2 (100%)	672.0 (441.23)
Total	70/123 (56%)	45.9 (110.50)	32/60 (53%)	33.6 (61.89)	38/63 (60%)	57.5 (141.76)

Abbreviations: ED, Emergency Department; SD, standard deviation
